# COVID-19, The Rule of Law and Democracy. Analysis of Legal Responses to a Global Health Crisis

**DOI:** 10.1007/s40803-022-00168-8

**Published:** 2022-03-15

**Authors:** Joelle Grogan

**Affiliations:** 1 Middlesex London, London, UK; 2 CEU Democracy Institute, Budapest, Hungary

## Abstract

The COVID-19 pandemic caused a severe strain on health systems globally, while simultaneously presenting a social, economic, legal, political, and regulatory challenge. Where the efficacy of pandemic laws adopted by governments are a matter of life and death, the urgency with which action needs to be taken during a pandemic creates a law-making environment which incentivises rapid action without scrutiny and the use of power without restraint. Under such conditions, adherence to the foundational values of democracy and the rule of law come under increased pressure if not threat. The demands of emergency provide a convenient guise and means of justification for the use of power which only serves to consolidate power within the executive to the detriment of the separation of powers and weakening of the institutions of liberal democracy. This article provides a preliminary analysis on how the global health crisis has affected the state of democracy and the rule of law. While the specific examples are drawn from across the globe to highlight common trends and concerns, specific highlight is given to the EU and its Member States. It offers an outlook on how to prepare for future emergencies by building on the lessons of the current one.

## Introduction

The COVID-19 pandemic has had a profound impact on governance globally. Where the efficacy of pandemic laws adopted by governments are a matter of life and death, the urgency with which action needs to be taken during a pandemic creates a law-making environment which incentivises rapid action without scrutiny and the use of power without restraint. Under such conditions, a high level of adherence to democratic and rule of law principles—common and foundational values of the European Union and its Member States^1^—can be undermined. While no state has proven immune from criticism for the manner in which law and executive power was used in response the COVID-19 pandemic, the health emergency may yet prove to have been a catalyst in some EU Member States for accelerated rule of law backsliding.^2^ In these states, the exigencies of emergency provide a convenient guise and means of justification for the use of power which only serves to consolidate power within the executive to the detriment of the separation of powers and weakening of the institutions of liberal democracy.

The domination of the executive in decision-making during an emergency is neither surprising nor inherently a concern where they are subject to effective safeguards and democratic controls. However, in a concerning number of states, executives dominated decision-making over pandemic response to the extent that they appeared to ‘rule-by-decree’, operating with such a latitude of discretion as to act ‘above the law’. Echoing the concerns of many international organisations and NGOs,^3^ the 2021 V-Dem report decried the global trend towards democratic decline through increasing autocratisation of states through the use of the pandemic threat by certain governments to consolidate power within the executive.^4^ Such negative practice from a rule of law perspective was exacerbated where the separation of powers was weakened by the marginalisation of parliaments,^5^ and the minimisation of judicial scrutiny.^6^ The longer that pandemic governance extends, the more normalised such concentrated use of power and extreme measures limiting rights and civil liberties will become: a deeply troubling ‘new normality’ in a post-pandemic world.

Building on the wealth of research published in the “Power and the COVID-19 Pandemic” symposium, which analysed the impact of the pandemic on legal systems in 64 countries, including 26 EU Member States,^7^ this article provides a preliminary analysis on how the global health crisis affected the state of democracy and the rule of law. While examples are drawn from across the globe to highlight common trends and concerns, specific highlight is given to the EU and its Member States and the actions they took over the course of the first 18 months of pandemic.

In Sect. 2, this article explores the question of decision-making and democracy in the context of the global health emergency. It identifies forms of control and influence over decision-making, and interrogates potential democratic concerns which have arisen as a consequence. It then examines how pandemic governance exacerbated tensions at different levels of government; highlighting both the strengths and limitations of centralised and decentralised decision-making in emergency situations. It finally focuses on democratic discourse and engagement during pandemic, including the challenges to elections and the difficulties of ‘misinformation’ which have pervaded discourse on the most appropriate action to be undertaken during the COVID-19 pandemic.

Section 3 focuses on the implications for the rule of law, framed by principles which form its foundation—legality, legal certainty, accountability through parliamentary oversight and judicial review, the prohibition of arbitrariness, and equality before the law. It highlights common challenges to ensuring a high level of adherence to the rule of law in times of emergency, but advocates the position that the global health emergency should not negate the importance of adherence to the rule of law; and that such adherence could guide the most effective responses to emergency.

Finally, Sect. 4 offers an outlook on how to prepare for future emergencies by building on the lessons of the current one. It draws keys lessons and recommendations from the best, and most concerning, examples of pandemic governance.

## Democracy

### The Decision-Makers and the Question of Democratic Deficit

In such a complex, polycentric and multifaceted emergency as the global COVID-19 pandemic, a central question was *who* should be the dominant decision-maker, and *how* should decisions be made. In a democracy, the allocation of scare state resources—particularly health services and fiscal support—ought to be determined by the elected with ultimate accountability of their decisions to the electorate. However, where an emergency situation typically creates an unexpected situation demanding complex calculations paired with the need for urgent decision-making, those elected prior to the advent of the COVID-19 pandemic were not elected with governance of such an emergency in mind. Just as existing legal frameworks may not have accounted for the unexpected exigencies of a global pandemic,^8^ governments and legislatures were largely unprepared. While globally, states have diverged in their responses to the pandemic, both in levels of restriction imposed on populations, and in abstract ideological terms on the level of threat that the virus posed, it is nonetheless possible to observe common and distinct trends of influence over decision-making practices worldwide.

In the absence of a clear identification of the most effective courses of action, many governments have sought to rely on health expertise in the decision-making and design of pandemic measures. The creation of expert advisory groups, with different degrees of influence over the creation of pandemic measures, was a common feature. For example, in South Africa with the formation of Ministerial Advisory Committees (MAC) on COVID-19, social and behavioural change, and vaccines. While there was no obligation to follow the MACs opinion, the government indicated that it followed the advice 95% of the time, and—in response to criticism—made the advice public in support of transparency.^9^ However, further criticism was levelled where the government restricted disapproval of its pandemic management, even from within the MACs.^10^

Reliance on expertise does not immunise decision-making from democratic concerns, particularly where meetings between experts and decision-makers are held in camera behind closed doors, the rationale for measures is opaque, unpublished, or produced by only a limited number of expert opinions. Technocratic governance can also raise democratic concerns, where decisions impact not only the allocation of state resources, but also fundamental rights and freedoms (for example, when to restrict movement, close borders, or place the state under national or localised lockdowns) appear to be determined not by elected officials, but by unelected experts. In the Netherlands,^11^ the government response was primarily led by the advice of epidemiological experts, who directed the introduction of measures restricting constitutional freedoms including the restriction of gatherings for protest or religious practice. It can appear that such experts are democratically unaccountable, yet are significantly influencing, if not ultimately determining, decisions which ought to be.^12^ This becomes all the more concerning where the rationale for measures is unpublished or otherwise unavailable for scrutiny. While the accountability of governments is ultimately to the electorate, the responsibility (and accountability) of experts is to their field and their profession: the reasoning of both should be open to informed scrutiny.

A further criticism of technocratic pandemic governance was the relative poverty of influence of expertise beyond virologists and epidemiologists.^13^ The pandemic and measures adopted in response to it negatively impacted more than public health, but also, inter alia, liberty, access to justice, education, livelihood, property, and privacy. Tackling not only the disease, but the ‘shadow pandemics’ of e.g. domestic violence and rising poverty which spread as a consequence of measures taken in response to it, demands more than (albeit essential) health expertise. A lesson for future pandemic emergency response is that diverse types of expertise should be harnessed to advise political decision-makers, though, it must be acknowledged that such consultation may more likely be able to inform medium- to longer-term response and recovery, rather than the immediate response to an emerging crisis.

By contrast to decisions driven primarily by public health interests, in a number of states globally, the pandemic crisis was treated as a security crisis. This securitiation often correlated with the militarisation of pandemic response, for example, where military personnel have replaced medical professionals in decision-making processes.^14^ In Hungary, instead of relying primarily on democratic institutions, the military became the source of both authority when placed in senior positions in hospitals as well as telecommunications, transport and healthcare companies, but also enforcement where charged with implementing pandemic measures including curfew.^15^ The justification often offered for the militarisation of pandemic response was increased efficiency and capacity. During pandemic, military structures have supported civilian-led responses, but in some states (e.g. the Philippines, and Indonesia) it primarily led it. A consequent ‘militarised mentality’ can push executive choice towards “expansive, unaccountable emergency law”^16^ reminiscent of dictatorship. Under such a regime, ordinary civilian administration can become marginalised, underfunded and dysfunctional. It also severely risks undermining or otherwise delegitimising a democratic response, and can serve as a weight against democratisation processes already weakened by a heavy-handed pandemic response.^17^ Under a militarised leadership, mere obedience, rather than offering justified reasoning and clear guidelines, is the strategy for public compliance.^18^ Such militarised response also often goes hand-in-hand with autocratic or autocratising governance.

An assumption widely circulated through the early response to the pandemic was that autocratic states were able to respond to an emergency situation more effectively than democracies that respect constitutional restraints on executive power.^19^ Some states too have propagated politicised dichotomies of effective pandemic management being a choice between protecting public health or human rights—often favouring the former to the detriment of the latter.^20^ However, overconfidence in autocratic control of a situation—especially to the exclusion of expert advice—can easily produce drift and complacency,^21^ as evidenced to devastating and tragic effect in India^22^ and Malaysia.^23^

A further concerning trend is weak or no pandemic governance altogether: for example, chaotic decision-making indicating that executives are uncertain about policy choices or the objectives to be pursued bolsters the perception that executive reasoning was not based on any sound scientific, economic or political rationale. Reliance on pseudo-scientific claims, or those with no scientific basis as in Brazil^24^ and Pakistan^25^ correlated with the absence of central policy control, if not outright refusal to acknowledge the threat, and connected with “policy inertia, poor messaging, and inconsistent enforcement.”^26^ Where the governance is “chaotic, uncoordinated, inconsistent, and unpredictable”,^27^ public compliance is abandoned, and public trust is demolished and the capacity of states to effectively respond to pandemic is diminished.

### Tensions in Multilevel Governance

At the national level, a tension that was exposed during the pandemic was between federal executives and regional or state governments particularly where it stemmed from uncertainty over the division of competences. For federal powers “to assume power is easier than to return power”,^28^ marking a reluctance in returning competences to devolved administrations when the urgency of the situation was relieved. Where political division exists—particularly ideological divisions concerning the threat of the virus—this can further undermine collective effort at control. Potentially motivated by political desire to demonstrate autonomy, the Tigray region of Ethiopia departed from following federal government action, which ultimately culminated in a military confrontation,^29^ while anti-epidemic rhetoric found support in regions of Ukraine opposing the central government’s messaging.^30^ Uncertainty and a lack of coordination at federal level, or institutional incapacity to regulate regions undermines the efficacy of a national pandemic response as it necessitates local leaders to assume control without centralised or coordinated measures. The consequent intra-state inconsistency is unsustainable in the long term.

The Swedish model of decentralised powers and quasi-autonomous administrative agencies enabled local expertise and fast response, but nevertheless revealed areas which could not be regulated under existing provisions (for example, businesses could not be legally obligated to require employees to work remotely).^31^ Even where powers were speedily introduced at national level to coordinate the country’s response, they were not used. Opposing a trend of executive aggrandizement, criticism centred on whether the central government had *under* exercised its powers. However, the country must nevertheless be lauded for a culture of consensus, the diffusion of power, and supporting localised expertise.^32^ Germany was praised for intergovernmental dialogue, however, these discussions were held privately and regulations were still introduced through executive decree rather than through federal or *Länder* (state) parliaments.^33^ In Australia, the model of a National Committee—built on the principles of confidentiality and solidarity among committee members—meant that executive decisions were opaque.^34^ A positive outcome does not necessarily mean the best democratic processes were  in place.

Where national policy is uncoordinated or poorly-managed, proactive local government can serve as a fast-acting, locally responsive body—particularly where the local governments coordinate civil society and private bodies and enjoy high levels of trust among local communities. However, localism can ultimately be hampered by a lack of resources, poor institutional management, or (as in the Philippines) “the dominance of patronage politics, corruption and other problems”.^35^ A lesson for future approaches should be to aim to achieve a balance between the benefits of local knowledge (see, for example, the practices of Austria,^36^ and Estonia^37^), expertise and adaptation, the resource and coordinating capacity of national governments, and the expertise and information-gathering role of multilateral institutions. Ultimately, what is most effective may be a nationally coordinated response which is informed by intergovernmental dialogue, and which supports local innovation, expertise and response.

With regards to the European Union, it played a very limited role with regard to pandemic response—hampered by the limited competence it can exercise in the field of public health.^38^ The Treaties do not provide explicit provisions for response to a health emergency. The Common Security and Defence Policy under Article 43 Treaty on European Union [TEU] refers to provisions for crisis management but within the context of conflict prevention and peace-keeping operations, and was not interpreted to provide competence for action in a pandemic. However, or some more relevance, the solidarity clause under Article 222 Treaty on the Functioning of the European Union [TFEU] provides for the EU and EU countries to act jointly to provide assistance to another EU Member State which is the victim of a natural or man-made disaster. Article 168 TFEU provides that the Union is to complement and support national health policies, and also to encourage cooperation between Member States, in full respect of the responsibilities of Member States for the definition of their health policies and for the organisation, management and delivery of health services and medical care. This limited competence reflects the preferences of Member States,^39^ and the lack of political will to integrate in this area.^40^

The limited nature of competences arguably led to the consequent limited institutional capacity and lack of resourcing to lead a coordinated response to the pandemic. However, as argued by Pacces and Weimer, this may not be a sufficient reason to bolster competences: the EU has neither legal nor “sufficiently strong democratic-political authority”^41^ to take leadership of the COVID-19 response. The complexity of the organisation of national health and social care systems, which necessarily rely on political decision-making in the allocation of state resources, make national or regional governments better placed than the EU in terms of democratic accountability. The negative consequence, as evidenced within the EU where national governments primarily if not exclusively led responses, was the diverse and sometimes incompatible strategies adopted among EU Member States creating problematic consequences for the maintenance of free movement across borders.

### Elections, Information and Democratic Discourse

A challenge faced by a number of states was the question of how, and if, to run elections where pandemic measures restricting movement and gatherings made both campaigning, and the holding of elections, challenging. At the outset of the pandemic, states in the middle of election cycles were faced with the challenges of governing in a crisis without an elected government. In Ireland, an interim government adopted sweeping measures,^42^ while in Belgium a minority government was tasked with forming the initial response to the crisis.^43^ As pandemic extended, elections and referenda were cancelled or rescheduled as efforts were made to strike a balance between the protection of public health and the right to vote. However, in a number of states,^44^ this appeared to reflect less a concern for public health than political interest in maintaining the status quo. In Poland, the presidential elections were cancelled days before they were scheduled to take place with the announcement by the government that the Supreme Court would find such an election ‘unconstitutional’^45^ exposing “constitutional tragedy amid political farce”.^46^ Conversely, states that followed their election schedules, but failed to introduce any protective measures, forced the electorate to choose between their democratic right to vote, and the health of themselves, their family and their community.^47^

But even before elections, democratic discourse was deeply impacted the ‘pandemic of misinformation’^48^ encompassing not only proliferation of ‘fake news’ but also the increasingly controlled use of information by governments, and the lack of transparency behind government action. Brazil, China, Hungary, and Russia restricted or suspended the right to receive public health information or criminalised the spreading of ‘misinformation’ about public health. India’s (mis)reporting of the situation on the ground was buoyed by political encouragement to avoid ‘critical reporting’ and compounded by arrests of journalists.^49^ In Sri Lanka, there was no information concerning acquittals or convictions under the quarantine curfew provisions.^50^ In Pakistan “rather than recognizing the need for a more collaborative consensus-based approach to governing, the pandemic response was driven more by the battle of narratives.”^51^ Ideological objection to lockdowns by the Prime Minister led to a ‘lives versus livelihood’ standoff between federal and provincial government as regional governments locked down their states even while the Prime Minister dismissed such lockdowns as “anti-poor policy propagated by the elites”.^52^ In the US, political polarisation underlined the need for evidence-based politics rather than “faith, authority, partisanship, or wishful thinking”, and in the hyper-partisanship of US politics, science became a battleground.^53^

Poor response resulted in poor evaluation by the public, whereas successful management strengthened regimes. A failing in communication, inaccurate information, or an absence of reasoning, coupled with the lack of transparency and accountability—can infect and weaken public trust. Public trust, while not easily quantifiable, is arguably central to the most stable and effective governance and institutions. The legitimacy of government action is strongly connected with public access to information and understanding the justification of measures. However, this is not simply about access to information, or understanding, or even ‘trust’ in the science or experts advising government, but also in the larger sense of trust “built among citizen and between citizens and the state”.^54^

The government’s failure to provide information on the pandemic in Kenya provided the impetus for civil society to bridge the gap to provide a people-centred approach to responding to the pandemic.^55^ Such a move towards active community engagement (where national governance was absent or uncoordinated) was also seen in the Philippines where local government and communities helped fill economic and social gaps in protection, and provided information on the pandemic.^56^ Such public engagement and participation had net positive benefits and should be encouraged as good practice. As evidenced by Taiwan, “the key to prevent tyranny in pandemic control is a transparent and responsive political process in which citizen activism is a crucial part.”^57^ Even where the protection of public health may justify *short-term* limitation of political accountability through the legislature, there must be a robust commitment to public rationality through transparent decision-making processes as this is essential to democratic discourse and ultimately a health democratic system.

## Rule of Law

### Legality and Legal (Un)Certainty

The rule of law is ‘the backbone of modern constitutional democracies’,^58^ and considered a ‘prerequisite for any efficacious legal order’.^59^ Within the European Union, it is enshrined in Article 2 of the Treaty on European Union, and includes

“the principles of legality, implying a transparent, accountable, democratic and pluralistic process for enacting laws; legal certainty; prohibition of arbitrariness of the executive powers; effective judicial protection by independent courts, including of fundamental rights; separation of powers and equality before the law”.^60^

The rule of law is “a meta-principle with formal and substantive components which guide and constrain the exercise of public authority and protect against the arbitrary or unlawful use of public power” and “as a primary and transversal constitutional principle, [it] shares a substantial and mutually reinforcing relationship with democracy and respect for human rights”.^61^ Within the environment of the COVID-19 pandemic, however, adherence to this value was strained. The early stages of the pandemic were characterised by extreme uncertainty as to which were the most effective strategies to limit risk and exposure—particularly where the virality and forms of transmission were uncertain. However, even a year following the initial outbreak of the COVID-19 pandemic, measures continued to be introduced with little^62^ or no notice, published after they have come into force,^63^ or even applied without publication at all.^64^ The announcement of measures within hours of introduction or even with immediate effect left thousands—sometimes millions—stranded abroad, forced to internally migrate,^65^ or queue for hours to receive a permit giving permission to commute the next day.^66^ Measures were contradictory, and the public had to rely on governments to explain legal orders in press conferences as they were unclear on the page.^67^ Such legal uncertainty undermined the efficacy of the rules designed to limit infection and transmission of the virus, but also served to undermine coordinated efforts to ensure high levels of compliance with pandemic measures.

Where the principle of legality implies that governments should be bound by the limits of the law, and not on the basis of ‘mere discretion’, many states acted primarily *with* discretion which often rested on questionable legal bases.^68^ In some states, the law was “kept out of the way”^69^ of political actors: for example, in Sri Lanka the measures keeping millions under ‘quarantine curfew’ did not have a clear legal basis.^70^ Where constitutional provisions require that restrictions on constitutional rights must be based on express provision of statute, or through an act of Parliament, this raised concerns about legality where executives have instead relied on secondary legislation or government-made orders.^71^ In some cases, executive measures were retrospectively legalised where the legal basis for the measure was found to have been lacking, or even non-existent^72^ at the point it was ordered.

Laws pre-dating the modern era can be ‘outdated’ by modern democratic standards of accountability and oversight but were nevertheless relied upon. The unsuitability of reliance on such pre-existing ordinary legislation was highlighted in India^73^ and Cyprus,^74^ where colonial-era acts provided for arguably *too* much discretion for executive action without parliamentary oversight. Reliance on such provisions highlight two issues: first, a lack of appropriate legal frameworks for use during health emergencies, or second, even where arguably there *is* such provision—a political unwillingness to rely on it, due to (for example) higher standards of scrutiny, or less provision for discretion. By contrast, good practices can be identified in New Zealand: the government actively engaged in communication across a variety of media so that the population could understand the necessity of the measures adopted. In the early stages, the government and enforcement authorities were circumspect in their enforcement where the legality of the measures was uncertain—and have engaged widely and broadly when remedying any legal deficiency.^75^

### Accountability Through Parliamentary Oversight and Judicial Review

A majority of states’ responses were primarily (if not exclusively) led by the executive. This is unsurprising. The fast-changing situation of the pandemic necessitated an urgent response, and one which would often not have been possible through (comparatively longer) legislative processes. Such executive dominance of decision-making in response to the pandemic is not necessarily a concern, nor an indicator of democratic deconsolidation or abusive practices (just as the declaration of a state of emergency is not innately problematic^76^). However, the extended duration of such dominance should be treated with higher levels of scrutiny where it can create permanent changes to the legal system or a permanent shift in the balance of powers in favour of the executive or to the detriment of the separation of powers. The safeguard against such a permanent shift is the accountability of the executive through parliamentary oversight and effective judicial review.

Parliamentary oversight and judicial review provide an essential function in every democracy by guarding against the potential abuse of power by the executive. One of the most concerning trends identified early in the pandemic and persisting more than a year later was the marginalisation of the role of parliaments, as evidenced in *inter alia* Australia,^77^ Bulgaria,^78^ Colombia,^79^ Cyprus,^80^ India,^81^ and Iran.^82^ In some states, Parliaments had no role,^83^ or had been effectively suspended.^84^ While deference in an emergency served as justification for some of this lack of oversight, it also evidences pre-existing trends. In the UK, only five hours of parliamentary debate were allocated to pandemic measures under the Coronavirus Act 2020 in the year following its enactment.^85^ The Hungarian government, which had effectively become the EU’s first autocracy prior to COVID-19, capitalised on the situation of the pandemic to empower itself to all but rule by decree with limited possibility of accountability through either judicial review or parliamentary oversight.^86^

Such limited capacity for oversight fundamentally undermines the capacity of parliaments to provide oversight on the actions of government. The key point of emphasis on this point is not temporary suspension of certain forms of activity or oversight (for example, the limitation on parliamentary meetings, or the movement of parliamentary business into a virtual setting) but instead where it marks an ongoing and continued trend of marginalisation of parliaments. Again, the central point is of permanence of change which, for some states, was shown to be a continuation of trends prior to pandemic rather than in direct response to it.

Finland serves as an example of positive practice: immediate parliamentary scrutiny on the use of any emergency powers was required in the use of emergency powers, as well as engagement with external experts, including independent constitutional law experts, by the specialised Parliamentary committees.^87^ The country also maintained a “continued commitment to the restoration of normalcy as a lodestar for any use of emergency powers.”^88^ In Taiwan all control orders were subject to parliamentary oversight and must be submitted to Parliament “as soon as possible”.^89^ By contrast, in France, the new state of health emergency which was introduced in response to the pandemic provided no such obligation for executive and administrative authorities to send copies of their acts to Parliament.^90^

Even where it served as a catalyst for the most concerning developments, the pandemic also provided space for innovation in parliamentary practices. The government commitment in Singapore to promote the continued functioning of parliament and engagement with the public through live-streaming sessions, shows the potential and possibility for a “more robust form of democratic government”.^91^ These practices can and should be adopted: regular and mandated reporting to parliaments by ministers, the introduction of specialised oversight committees, and open and active engagement with the public through virtual sessions can manifestly improve democratic scrutiny and accountability.

Where parliamentary oversight is limited, however, either by design or through deference, courts have become in some instances the only institution with direct oversight of government action. In some extreme examples, the courts have found astonishing numbers of penalties imposed under pandemic measures to be groundless. For example, 90% of administrative proceedings in Ukraine were found to be in error,^92^ while in the UK,^93^ a cross-parliamentary group found that the offences related to infectious people under the Coronavirus Act 2020 were so misunderstood and wrongly applied that all criminal charges under the Act were incorrect.^94^

Issues were raised where there is little capacity for judicial scrutiny, for example where an ouster clause can effectively block review of laws, or where a government removes access to the courts through pandemic-mandated closures. In Bangladesh for instance, all courts were initially shut down by the government, without further consultation, though later e-courts were allowed to operate during the pandemic.^95^ Even where courts are not closed, excessive deference to government control had the potential to distort standards of judicial scrutiny. However, on one interpretation, such deference represented adherence to the ‘political question’ doctrine, acknowledging that democratic legitimacy on such issues comes from the legislature—not the courts, and implicitly recognised the lack of uniform standards based on scientific knowledge, or low awareness on the part of judges of how to weigh relevant epidemiological evidence to make informed decisions on matters of public health. It is possible too that the speed and pace of change left little capacity for courts to develop a consistent line of judicial reasoning. Nevertheless, dissenting opinions on major constitutional questions of the legitimacy of government action challenged judicial deference and majority reasoning, particularly where it had the effect of “watering down the principle of proportionality to mere rationality”.^96^

However, even where significant challenges were exposed in effective judicial protection and parliamentary oversight, the pandemic provided the opportunity for innovation-by-necessity, as the world embraced an approach of ‘techno-solutionism’ through virtual proceedings in parliaments and court hearings. These innovations were not without practical challenges for populations, as for example the lack of access to the internet can undermine the efficacy of online democratic engagement, or limit access to public services. These challenges are not without solutions, however, and innovations should be embraced.

### Prohibition of Arbitrariness of the Executive Powers

In evaluating government action, the focus should be on the *use*—not necessarily *form*—of law,^97^ with awareness of pre-existing national trends towards democratic deconsolidation and rule of law backsliding. In examining the *use* of law, and particularly the widespread reliance on emergency powers and executive orders, executive aggrandisement, or executive overreach^98^ became an identifiable reality in many states. Such centralisation of power in the executive can be to the detriment of constitutional safeguards against majoritarian rule, and often such power is exercised abusively *despite* safeguards.^99^ Where manifest, this abuse of emergency powers continues pre-existing trends^100^ towards global democratic regression.^101^ For example, a type of “executive arrogance” in the Czech Republic culminated in an unconstitutional declaration of a state of emergency by the government.^102^

In response to the concerns about executive dominance, legal uncertainty, and the lack of parliamentary oversight and control, a common theme across countries was to press for targeted legislation which addressed the pandemic, returning oversight and control to legislatures as well as providing a clear and limited legal basis for action. For example, as advocated for Colombia,^103^ such a legislative act would “resolve the questions of who can take measures that severely restrict the fundamental rights of citizens for the control of a pandemic and how such measures can be adopted.” Similar calls for parliamentary legislation were made in India and Bangladesh,^104^ particularly where such an act—or at least reform of pre-existing provisions—could provide a framework based on equality and transparency.

However, ‘Corona Acts’ have rarely, if ever, proven to be a panacea. Legislation introduced in the haste that emergency provokes can often suffer from legal deficiencies including vague and open-ended terms providing for the wide delegation of discretionary powers, and a lack of parliamentary oversight both at its promulgation, but also in its application.^105^ For example, the Swiss’ ‘COVID-19 Act’, was “too vague for ordinary legislation” and as emergency legislation, it represented a “form of empowerment vis-à-vis the government that lacks a constitutional basis”.^106^ Where such acts make permanent changes, they introduced a form of ‘emergency creep’ infecting ordinary law and governance beyond the pandemic.

These concerns were all the more manifest where ‘new’ states of [health] emergencies were created, but their use and limits were dictated by governments rather than constitutions. France allowed for the “deep and wide” restriction of rights but does not ostensibly introduce measures to tackle the pandemic which did not previously exist.^107^ In Bulgaria the question of when to introduce the new ‘state of epidemiological conditions’ was determined by the executive with uncertain definition and constraints, and which allowed for the limit of fundamental rights based on executive orders only.^108^

The experiences of Singapore,^109^ Taiwan^110^ and Hong Kong^111^ evidenced the importance of reform and preparation for the next pandemic by learning from past epidemiological events. Taiwan, despite being barred entry from entry to the World Health Organisation, and its close proximity to China, was considered paradigmatic of a ‘successful’ response to the pandemic by having both low mortality and infection rates coupled with the least restrictive measures in the world, and with relatively minimal disruption to government services, access to the courts, business and education. Reforms of the law and institutions, notably the Centre for Disease Control, enabled fast action as well as providing government with capacity to introduce measures for economic relief, compensation, and stimulus. A fast-acting government response, coupled with extensive and free public testing and tracing, meant that South Korea avoided implementing many of the more restrictive measures including lock-down, and did not declare a state of emergency.^112^ In echo of similar experience in Taiwan^113^ and Hong Kong,^114^ such action was enabled by reforms following the mistakes made during the initial responses to the MERS outbreak in 2015. A lesson for the post-pandemic environment is that legal reform will be necessary as states examine the efficacy and adequacy of their responses to the pandemic.

### Equality Before the Law

The pandemic exposed endemic social and structural weaknesses, as both the virus and measures taken in response to it most negatively impacted those already in a vulnerable position, including minority communities, women and children, asylum-seekers, the elderly, those with physical or mental disabilities, refugees and undocumented migrants. Domestic and gender-based violence spiked across the world. As Alice Donald and Phil Leach write^115^:

This dramatic increase results from a perfect storm of factors, which itself exemplifies the interdependence of human rights of all kinds. Restrictions on movement, economic insecurity, a decrease in police interventions, and the closure of courts and emergency services have emboldened perpetrators and aggravated the risks faced by women and girls.

Enforcement measures were more likely to be applied to already marginalised communities, and were applied in a discriminatory manner against already stigmatised groups including the disabled, minority religious communities, and LGBTQ+. Such arbitrary application is damaging. Inconsistency in the application of provisions showed political bias, as in Sri Lanka where the use of guidelines was to prevent protest by families of the disappeared, but was not applied to government events and private functions attended by officials. In Croatia^116^ and Kenya,^117^ disproportionately favourable treatment was shown to politically important events favourable to the ruling parties. Measures provided a cloak for targeted discrimination against protesters, political opponents and those who criticised government decision-making including doctors and journalists. In Turkey^118^ and Poland,^119^ peaceful protests were banned but political gatherings in support of government were praised and took place without restrictions being enforced.

## Conclusion

In preparing for the future, there are clear lessons for the reform of law and legal frameworks built on the values of the rule of law, and democracy.^120^ As argued elsewhere, the rule of law provides a “perimeter of legitimacy of the restrictive measures taken in response to the pandemic.”^121^ The pandemic exposed critical issues from a rule of law perspective: COVID-19 measures were often uncertain in their meaning, arbitrary in their application, and of questionable basis in the law. Oversight was often limited or lacking in many states, and the laws introduced during the pandemic risked causing permanent shifts in the balance of power towards the executive. These are trends that should be resisted. However, the pandemic also showed the potential for democratic innovation, and citizen engagement in decision-making processes, and in this way reveals a path forward to resist pressures towards rule of law backsliding and democratic decline.

Active political and democratic engagement across the widest and most diverse range of society is essential to (re)build public trust in governance and institutions and their capacity to manage crisis. Broader and more interdisciplinary expertise is needed to inform response to the ‘shadow’ pandemics which are parasitic on the COVID-19 health emergency. Collaboration and coordination across levels of government is more effective, but requires clear guidelines as to competence, capacity and communication. The pandemic created opportunities for the consolidation of democracy and innovation in the protection of rights, as well as the opportunity to prove the resilience of democratic institutions. Where it exposed and deepened endemic socio-economic inequalities, it also showed us how and why they must be remedied through global efforts and worldwide solidarity. Trust and solidarity are bedrocks of success but are hard earned – relying on commitments of the powerful and participation of the powerless.

## Footnotes

1 Article 2 Treaty on European Union. See e.g. Laurent Pech and Joelle Grogan (eds), ‘Unity and Diversity in National Understandings of the Rule of Law in the EU’ RECONNECT Working Paper 7.1 (May 2020) https://reconnect-europe.eu/publications/; and Laurent Pech and Joelle Grogan (eds), ‘Meaning and Scope of the EU Rule of Law’ RECONNECT Working Paper 7.2 (May 2020) https://reconnect-europe.eu/publications/.

2 See e.g. Dimitry Kochenov and Petra Bárd, ‘Rule of Law Crisis in the New Member States of the EU: The Pitfalls of Overemphasising Enforcement’ RECONNECT Working Paper No. 1 (July 2018); and Paul Blokker et al., ‘The democracy and rule of law crises in the European Union’ RECONNECT Working Paper 14.1 (April 2021).

3 See e.g. the World Justice Project Rule of Law Index 2021, available at: https://worldjusticeproject.org/our-work/research-and-data/wjp-rule-law-index-2021; IDEA International, *Global State of Democracy 2021*: *Building Resilience in a Pandemic Era* (International Institute for Democracy and Electoral Assistance, 2021) https://www.idea.int/gsod/; Sarah Repucci and Amy Slipowitz, Democracy under Lockdown: The Impact of COVID-19 on the Global Struggle for Freedom (Freedom House 2020); Abebe et al. (2021).

4 V-Dem Institute ‘Autocratization Turns Viral, Democracy Report 2021’ < https://www.v-dem.net/media/filer_public/74/8c/748c68ad-f224-4cd7-87f9-8794add5c60f/dr_2021_updated.pdf > 

5 See e.g. Cormacain and Bar-Siman-Tov (2020); and Griglio (2020).

6 See e.g. Baldwin et al. (2020).

7 The “Power and COVID-19 Pandemic” (2021) involved the contributions of over 100 experts, critically assessing the legal response to the COVID-19 pandemic in 64 countries worldwide in the year since the declaration of a global health emergency. The states within the symposium were: Australia, Austria, Bangladesh, Belgium, Brazil, Bulgaria, Canada, China, Colombia, Croatia, Cyprus, Czechia, Denmark, Ecuador, Egypt, Estonia, Ethiopia, Finland, France, The Gambia, Germany, Greece, Hong Kong, Hungary, Iceland, India, Indonesia, Iran, Ireland, Israel, Italy, Kenya, Latvia, Lebanon, Lithuania, Luxembourg, Malaysia, Mexico, Netherlands, New Zealand, Norway, Pakistan, Peru, the Philippines, Poland, Portugal, Romania, Russia, Singapore, South Africa, South Korea, Slovakia, Slovenia, Spain, Sri Lanka, Sweden, Switzerland, Taiwan, Thailand, Turkey, Ukraine, the UK, and the USA. Contributors were drawn from academia, civil society, NGOs, the judiciary and legal practice, and critically reflected on how their legal and political systems adapted to the pandemic, analysing the legal measures and use of powers from the perspective of democracy, the rule of law and human rights. The countries represented a diversity of states in terms of geography, population size and density, income levels, forms of governance, legal traditions, experience (or not) of previous pandemics, and COVID-19 infection and mortality rates. All EU Member States, with the exception of Malta, were analysed. See Grogan (2021b, a, c).

8 See infra Sect. 2.4.

9 Staunton and Labuschaigne (2021).

10 Ibid.

11 Julicher and Vetzo (2021).

12 See e.g. Lavazza and Farina (2020).

13 Grogan and Yamin (2022)

14 See e.g. Molloy et al. (2021).

15 Kovács (2021).

16 Tonsakulrungruang and Leelapatana (2021).

17 Molloy et al. (n 14).

18 See e.g. Ellaboudy (2021).

19 See e.g. Justin Esarey, 2021. ‘The Myth That Democracies Bungled the Pandemic’ The Atlantic (4 October 2021); and James Traub, ‘The Future Is Asian—but Not Chinese’ Foreign Policy 27 April 2020.

20 See e.g. Donald and Leach (2020); and Donald and Leach (2021a, b).

21 See Daly (2022).

22 Raj (2021).

23 Balasubramaniam (2021).

24 Meyer and Bustamante (2021).

25 Syed and Tariq-Ali (2021).

26 Ibid.

27 Hoque (2021); and Raj (n 22).

28 Uhlmann and Ammann (2021).

29 Ayele and Fessha (2021).

30 Petrov and Bernatskyi (2021).

31 Cameron and Jonsson-Cornell (2021).

32 Ibid.

33 Mangold (2021).

34 Rizzi and Tulich (2021).

35 Atienza (2021).

36 Lachmayer (2021).

37 Kiviorg and Margna (2021).

38 See Grogan (2021b, a, c).

39 Brooks (2020); see also Brooks and Geyer (2020).

40 Pernhagen et al. (2020).

41 Pacces and Weimer (2020).

42 Doyle (2021).

43 Ridder (2021).

44 See e.g. Chile: Cofre (2020).

45 Kustra-Rogatka (2020).

46 Ibid.

47 See the debates in Landman and Splendore (2020).

48 See Tagliabue et al. (2020).

49 Raj (n 22).

50 Ganeshathasan (2021).

51 Syed and Tariq-Ali (n 25).

52 ibid.

53 Graber (2021)

54 Jasanoff and Hilgartner (2021). See also Sheila Jasanoff, Stephen Hilgartner et al., ‘Comparative Covid Response: Crisis, Knowledge, Politics Interim Report’ Harvard Kennedy School (January 2021).

55 Were et al. (2021).

56 Atienza (n 35).

57 Chang and Lin (2021).

58 European Commission, *A New Framework to strengthen the Rule of Law* (Communication) COM (2014) 158.

59 Margaret Jane Radin, ‘Reconsidering the Rule of Law’ (1989) BULJ 781, 783.

60 European Commission, *Further Strengthening the Rule of Law within the Union* (Communication) COM (2019) 163 Final 1.

61 Laurent Pech and Joelle Grogan (eds), ‘Meaning and Scope of the EU Rule of Law’ RECONNECT Working Paper 7.2 (May 2020) https://reconnect-europe.eu/publications/.

62 Bardutzky and Zagorc (2021).

63 Doyle (n 42).

64 Koroteev (2021).

65 See e.g. Hoque (n 27).

66 Tonsakulrungruang and Leelapatana (n 16).

67 Bardutzky and Zagorc (n 62).

68 In the context of EU Member States, see Joelle Grogan, 'Extraordinary or Extralegal Responses? COVID-19 and the Rule of Law in Europe' (Democracy Reporting International 2021).

69 As in Sri Lanka, Ganeshathasan (n 50).

70 Ibid.

71 For example, as in Poland: Jaraczewski (2021).

72 Lauta (2021).

73 See Raj (n 22).

74 Kombos (2021).

75 Knight (2022).

76 Grogan (2020).

77 Rizzi and Tulich (2021).

78 Vassileva (2021).

79 Hoyos-Ceballos and Gaviria-Mira (2021).

80 Kombos (n 74).

81 Raj (n 22).

82 Darian (2021).

83 See e.g. Staunton and Labuschaigne (n 9).

84 See e.g. Balasubramaniam (n 23).

85 Ruth Fox, Meg Russell, Ronan Comacain and Joe Tomlinson, ‘The marginalisation of the House of Commons under Covid has been shocking; a year on, Parliament’s role must urgently be restored’ (*Hansard Society*, 21 April 2021) https://www.hansardsociety.org.uk/publications/briefings/the-marginalisation-of-the-house-of-commons-under-covid-has-been-shocking-a.

86 Kovács (n 15).

87 Martin Scheinin, ‘Finland's Success in Combating Covid-19: Mastery, Miracle or Mirage?’ in Grogan and Donald (n 75).

88 Scheinin (2021).

89 Chang and Lin (n 57).

90 Basilien-Gainche (2021).

91 Chua and Neo (2021).

92 Petrov and Bernatskyi (n 30).

93 See Grogan (2021b, a, c).

94 House of Commons and House of Lords Joint Committee on Human Rights, *Fourteenth Report of Session 2019–21: The Government response to covid-19: fixed penalty notices* HC 1364, HL Paper 272 (27 April 2021).

95 Hoque (n 27).

96 Selanec (2021).

97 Grogan (2020).

98 Pozen and Scheppele (2020).

99 See e.g. Selanec (n 96); and Atienza (n 35).

100 Vedaschi (2021).

101 See e.g. V-Dem Institute ‘Autocratization Turns Viral, Democracy Report 2021’ https://www.v-dem.net/media/filer_public/74/8c/748c68ad-f224-4cd7-87f9-8794add5c60f/dr_2021_updated.pdf.

102 Vikarská (2021).

103 Julián and Esteban (2020).

104 Hoque (n 27); and Raj (n 22).

105 See e.g. and Selanec (n 96).

106 Felix and Odile (2021).

107 Basilien-Gainche (n 90).

108 Vassileva (n 78).

109 Neo and Chua (n 91).

110 Chang and Lin (n 57).

111 Pui-yin (2021).

112 Lee and Kim (2021).

113 Chang and Lin (n 57).

114 Lo (n 111).

115 Donald and Leach (2021a, b).

116 Selanec (n 96).

117 Were et al. (n 55).

118 Çalı and Turkut (2021).

119 Jaraczewski (n 71).

120 See further policy recommendations: Joelle Grogan and Alice Donald, ‘Policy paper on the implications of COVID-19 Insights into State Governance and the Rule of Law, Human Rights and Good Governance during the COVID-19 Pandemic’ RECONNECT Policy Paper (January 2022). https://reconnect-europe.eu/wp-content/uploads/2022/01/D11.2.pdf

121 Grogan and Beqiraj (2021).

## References

[CR1] Abebe A et al (2021) Annual review of constitution building 2020. International IDEA. 10.31752/idea.2021.102

[CR2] Atienza MEL (2021) The Philippines a year under lockdown: continuing executive dominance, threats to democracy, and unclear pandemic response. VerfBlog. 2021/4/26. https://verfassungsblog.de/the-philippines-a-year-under-lockdown/. 10.17176/20210426-173702-0

[CR3] Ayele Z, Fessha Y (2021) COVID-19 in Ethiopia: a year in review. VerfBlog. 2021/4/20. https://verfassungsblog.de/covid-19-in-ethiopia-a-year-in-review/. 10.17176/20210420-185012-0

[CR4] Balasubramaniam RR (2021) COVID-19: Malaysia’s fragile constitutional democracy. VerfBlog. 2021/3/02. https://verfassungsblog.de/covid-19-malaysias-fragile-constitutional-democracy/. 10.17176/20210302-154032-0

[CR5] Baldwin JM, Eassey JM, Brooke EJ (2020) Court operations during the COVID-19 pandemic. Am J Crim Just 45:743–758. 10.1007/s12103-020-09553-1 10.1007/s12103-020-09553-1 PMC7354363 32837170

[CR6] Bardutzky S, Zagorc S (2021) Slovenia: second wave of challenges to constitutionalism. VerfBlog. 2021/3/19. https://verfassungsblog.de/slovenia-second-wave-of-challenges-to-constitutionalism/. 10.17176/20210319-151656-0

[CR7] Basilien-Gainche M-L (2021) French response to COVID-19 crisis: rolling into the deep*. VerfBlog. 2021/3/18. https://verfassungsblog.de/french-response-to-covid-19-crisis-rolling-into-the-deep/. 10.17176/20210318-154150-0

[CR8] Brooks E, de Ruijter A, Greer SL (2020) Covid-19 and European union health policy: from crisis to collective action. In: Vanhercke B, Spasova S, Fronteddu B (eds) Social policy in the European Union: state of play 2020. European Trade Union Institute

[CR9] Brooks E, Geyer R (2020) The development of EU health policy and the Covid-19 pandemic: trends and implications. J Eur Integr 42:1057–1076

[CR10] Çalı B, Turkut E (2021) Year one: reflections on turkey’s legal responses to the COVID-19 pandemic. VerfBlog. 2021/3/16. https://verfassungsblog.de/year-one-reflections-on-turkeys-legal-responses-to-the-covid-19-pandemic/. 10.17176/20210316-154147-0

[CR11] Cameron I, Jonsson-Cornell A (2021) COVID-19 in Sweden: a soft power approach. VerfBlog. 2021/2/24. https://verfassungsblog.de/covid-19-in-sweden-a-soft-power-approach/. 10.17176/20210224-154142-0

[CR12] Chang W-C, Lin C-Y (2021) Legislative/judicial deference versus NGOs/citizens activism: Taiwan’s successful fight against Covid-19. VerfBlog. 2021/4/21. https://verfassungsblog.de/legislative-judicial-deference-versus-ngos-citizens-activism-taiwans-successful-fight-against-covid-19/. 10.17176/20210421-101205-0

[CR13] Chua S, Neo JL (2021) COVID-19 as an opportunity for democratic consolidation?: law, politics, and citizen-state interactions in Singapore. VerfBlog, 2021/2/24. https://verfassungsblog.de/covid-19-as-an-opportunity-for-democratic-consolidation/. 10.17176/20210224-154044-0

[CR14] Cofre L (2020) Chile and COVID-19: a constitutional authoritarian temptation. VerfBlog. 2020/5/19. https://verfassungsblog.de/chile-and-covid-19-a-constitutional-authoritarian-temptation/. 10.17176/20200519-133659-0

[CR15] Cormacain R, Bar-Siman-Tov I (2020) Legislatures in the time of Covid-19. Theory Pract Legis 8(1–2):3–9. 10.1080/20508840.2020.1816017

[CR16] Daly TG (2022) The pandemic and the future of global democracy. In: Grogan J, Donald A (eds) Routledge Handbook of Law and the COVID-19 Pandemic. Routledge

[CR17] Darian MT (2021) Iran’s COVID-19 response: who calls the shots? VerfBlog. 2021/3/12. https://verfassungsblog.de/irans-covid-19-response-who-calls-the-shots/. 10.17176/20210312-153956-0

[CR18] De Ridder M (2021) Belgium’s Accordion Response to COVID-19. VerfBlog. 2021/3/10. https://verfassungsblog.de/belgiums-accordion-response-to-covid-19/. 10.17176/20210310-154152-0

[CR19] Donald A, Leach P (2020) Human rights—the essential frame of reference in the global response to COVID-19. VerfBlog. 2020/5/12. https://verfassungsblog.de/human-rights-the-essential-frame-of-reference-in-the-global-response-to-covid-19/. 10.17176/20200512-133728-0

[CR20] Donald A, Leach P (2021a) Human rights and COVID-19: forging recovery after a pandemic of abuses? VerfBlog. 2021a/4/10. https://verfassungsblog.de/human-rights-and-covid-19-forging-recovery-after-a-pandemic-of-abuses/. 10.17176/2021a0410-101028-0

[CR21] Donald A, Leach P (2021b) Human rights and COVID-19: forging recovery after a pandemic of abuses? VerfBlog. 2021b/4/10. https://verfassungsblog.de/human-rights-and-covid-19-forging-recovery-after-a-pandemic-of-abuses/. 10.17176/2021b0410-101028-0

[CR22] Doyle O (2021) Pandemic response as accentuation of existing characteristics: vague requirements and executive dominance in Ireland. VerfBlog. 2021/3/17. https://verfassungsblog.de/pandemic-response-as-accentuation-of-existing-characteristics-vague-requirements-and-executive-dominance-in-ireland/. 10.17176/20210317-154047-0

[CR23] Ellaboudy A (2021) When emergency is permanent, what else could be done?: a year of sanitary crisis in Egypt. VerfBlog. 2021/4/15. https://verfassungsblog.de/when-emergency-is-permanent-what-else-could-be-done/. 10.17176/20210415-101236-0

[CR24] Felix U, Odile A (2021) Switzerland and the COVID-19 pandemic: a look back and a look into the future. VerfBlog. 2021/3/01. https://verfassungsblog.de/switzerland-and-the-covid-19-pandemic-a-look-back-and-a-look-into-the-future/. 10.17176/20210301-154022-0

[CR25] Ganeshathasan L (2021) Sri Lanka’s Legal Response to COVID-19: past trends and future prospects. VerfBlog. 2021/3/23. https://verfassungsblog.de/sri-lankas-legal-response-to-covid-19-past-trends-and-future-prospects/. 10.17176/20210324-031258-0

[CR26] Graber MA (2021) COVID-19, the United States and Evidence-Based Politics. VerfBlog. 2021/4/14. https://verfassungsblog.de/covid-19-the-united-states-and-evidence-based-politics/. 10.17176/20210414-101222-0

[CR27] Griglio E (2020) Parliamentary oversight under the Covid-19 emergency: striving against executive dominance. Theory Pract Legis 8(1–2):49–70. 10.1080/20508840.2020.1789935

[CR28] Grogan J (2020) States of Emergency. Eur J Law Reform 338–354

[CR29] Grogan J (2021a) The Limited Role of the European Union in the Management and Governance of the COVID-19 Pandemic’. Int Organ Law Rev 18:480

[CR30] Grogan J (2021b) A government (Un)Governed?: examining the UK’s Legal Responses to the COVID-19 Pandemic. VerfBlog. 2021c/5/07. https://verfassungsblog.de/a-government-ungoverned/. 10.17176/2021c0507-111655-0

[CR31] Grogan J (2021c) Power and the COVID-19 Pandemic—introduction & list of contributions. VerfBlog. 2021b/2/22. https://verfassungsblog.de/power-and-the-covid-19-pandemic/. 10.17176/2021b0222-154018-0

[CR32] Grogan J, Beqiraj J (2021) Rule of law as a perimeter of legitimacy for COVID-19 responses. VerfBlog. 2021/4/17. https://verfassungsblog.de/rule-of-law-as-a-perimeter-of-legitimacy-for-covid-19-responses/. 10.17176/20210417-101033-0

[CR33] Grogan J, Yamin AE (2022) A functionalist approach to analyzing legal responses to COVID-19 across countries: comparative insights from two global symposia. In: Cohen IG, Gluck A, Kraschel K, Shachar (eds) COVID-19 and the law: disruption, impact and legacy. CUP

[CR34] Hoque R (2021) Bangladesh’s COVID-19 year in review. VerfBlog. 2021/3/25. https://verfassungsblog.de/bangladeshs-covid-19-year-in-review/. 10.17176/20210325-151610-0

[CR35] Hoyos-Ceballos E, Gaviria-Mira J (2021) Dealing with the pandemic: a stress test for Colombian political institutions. VerfBlog. 2021/3/05. https://verfassungsblog.de/dealing-with-the-pandemic-a-stress-test-for-colombian-political-institutions/. 10.17176/20210305-154056-0

[CR36] Jaraczewski J (2021) The new normal?—emergency measures in response to the second COVID-19 Wave in Poland. VerfBlog. 2021/3/24. https://verfassungsblog.de/the-new-normal-emergency-measures-in-response-to-the-second-covid-19-wave-in-poland/. 10.17176/20210324-151409-0

[CR37] Jasanoff S, Hilgartner S (2020) A stress test for politics: insights from the comparative Covid Response Project (CompCoRe) 2020. VerfBlog, 2021/5/11. https://verfassungsblog.de/a-stress-test-for-politics-insights-from-the-comparative-covid-response-project-compcore-2020/. 10.17176/20210511-091104-0

[CR38] Julián G-M, Esteban H-C (2020) Pandemic and executive powers in Colombia: a problem and a modest proposal. VerfBlog. 2020/4/17. https://verfassungsblog.de/pandemic-and-executive-powers-in-colombia-a-problem-and-a-modest-proposal/. 10.17176/20200417-182608-0

[CR39] Julicher M, Vetzo M (2021) COVID-19 in the Netherlands: of changing tides and constitutional constants. VerfBlog. 2021/4/22. https://verfassungsblog.de/covid-19-in-the-netherlands-of-changing-tides-and-constitutional-constants/. 10.17176/20210422-101345-0

[CR40] Kiviorg M, Margna P (2021) COVID-19 in Estonia: a year in review. Verfblog. 2021/3/12. https://verfassungsblog.de/covid-19-in-estonia-a-year-in-review/

[CR41] Knight DR (2022) Accountability through dialogue: New Zealand’s Experience during the COVID-19 Pandemic. In: Grogan J, Donald A (eds) Routledge handbook on law and the COVID-19 Pandemic. Routledge. https://ssrn.com/abstract=3908769 or 10.2139/ssrn.3908769

[CR42] Kombos C (2021) Constitutional Improvisation and Executive Omnipotence: the Cypriot Handling of the Pandemic. VerfBlog. 2021/3/02. https://verfassungsblog.de/constitutional-improvisation-and-executive-omnipotence-the-cypriot-handling-of-the-pandemic/. 10.17176/20210302-154130-0

[CR43] Koroteev K (2021) A year of zeros? Legal responses to the COVID-19 pandemic in Russia. VerfBlog. 2021/3/01. https://verfassungsblog.de/a-year-of-zeros-legal-responses-to-the-covid-19-pandemic-in-russia/. 10.17176/20210301-153949-0

[CR44] Kovács K (2021) Hungary and the pandemic: a pretext for expanding power. VerfBlog. 2021/3/11. https://verfassungsblog.de/hungary-and-the-pandemic-a-pretext-for-expanding-power/. 10.17176/20210311-154209-0

[CR45] Kustra-Rogatka A (2020) Between constitutional tragedy and political farce: on the postponement of the presidential elections in Poland. VerfBlog, 2020/5/15. https://verfassungsblog.de/between-constitutional-tragedy-and-political-farce/. 10.17176/20200516-013414-0

[CR46] Lachmayer K (2021) Muddling through mutation times or the return of federalism in Austria: from Covid-19 response to the vaccination campaign. Verfblog 2021/5/8. https://verfassungsblog.de/muddling-through-mutation-times-or-the-return-of-federalism-in-austria/

[CR47] Landman T, Di L, Splendore G (2020) Pandemic democracy: elections and COVID-19. J Risk Res 23(7–8):1060–1066. 10.1080/13669877.2020.1765003

[CR48] Lauta KC (2021) The eternal emergency? Denmark’s Legal Response to COVID-19 in review. VerfBlog. 2021/3/22. https://verfassungsblog.de/the-eternal-emergency-denmarks-legal-response-to-covid-19-in-review/. 10.17176/20210322-151511-0

[CR49] Lavazza A, Farina M (2020) The role of experts in the covid-19 pandemic and the limits of their epistemic authority in democracy. Front Public Health 8:356. 10.3389/fpubh.2020.00356 32760690 10.3389/fpubh.2020.00356 PMC7372112

[CR50] Lee S, Kim T-H (2021) South Korea’s combating COVID-19 under the rule of law. VerfBlog. 2021/4/08. https://verfassungsblog.de/south-koreas-combating-covid-19-under-the-rule-of-law/. 10.17176/20210408-172700-0

[CR51] Mangold AK (2021) Germany and COVID-19: a most eventful year. VerfBlog. 2021/4/07. https://verfassungsblog.de/germany-and-covid-19-a-most-eventful-year/. 10.17176/20210407-170939-0

[CR52] Meyer EPN, Bustamante T (2021) COVID-19 in Brazil: a sick constitutional democracy. VerfBlog. 2021/2/22. https://verfassungsblog.de/covid-19-in-brazil-a-sick-constitutional-democracy/. 10.17176/20210222-153853-0

[CR53] Molloy S, Bell C, Welikala A, Houlihan E, Zulueta-Fülscher K (2021) Emergency law responses and conflict-affected states in transition. VerfBlog. 2021/3/13. https://verfassungsblog.de/emergency-law-responses-and-conflict-affected-states-in-transition/. 10.17176/20210313-153941-0

[CR54] Pacces AM, Weimer M (2020) from diversity to coordination: a European approach to COVID-19. Eur J Risk Regul 11:283

[CR55] Pernhagen KP, de Ruijter A, Flear ML (2020) Tamara Hervey and Alexia Herwig ‘more competences than you knew? The Web of Health Competence for European Union Action in Response to the Covid-19 Outbreak. Eur J Risk Regul 297–306

[CR56] Petrov R, Bernatskyi B (2021) Pandemic and fragile government: a year of COVID-19 fatigue and disorder in Ukraine. VerfBlog. 2021/3/04. https://verfassungsblog.de/pandemic-and-fragile-government-a-year-of-covid-19-fatigue-and-disorder-in-ukraine/. 10.17176/20210304-154007-0

[CR57] Pozen D, Scheppele KL (2020) Executive underreach, in pandemics and otherwise. Am J Int Law 114(4):608–617

[CR58] Pui-yin L (2021) Surviving executive-led pandemic control in executive-led Hong Kong: a year in review. VerfBlog. 2021/3/03. https://verfassungsblog.de/surviving-executive-led-pandemic-control-in-executive-led-hong-kong/. 10.17176/20210303-154036-0

[CR59] Raj TK (2021) COVID-19 and the crisis in Indian democracy. VerfBlog. 2021/2/26. https://verfassungsblog.de/covid-19-and-the-crisis-in-indian-democracy/. 10.17176/20210226-154112-0

[CR60] Rizzi M, Tulich T (2021) The Australian response to COVID-19: a year in review. VerfBlog. 2021/2/22. https://verfassungsblog.de/the-australian-response-to-covid-19-a-year-in-review/. 10.17176/20210222-153951-0

[CR61] Scheinin M (2021) Finland: Soft measures, respect for the rule of law, and plenty of good luck: Overview of Legal and Political Response and Adaptation to COVID-19. VerfBlog, 2021/2/23. https://verfassungsblog.de/finland-soft-measures-respect-for-the-rule-of-law-and-plenty-of-good-luck/. 10.17176/20210223-154147-0

[CR62] Selanec NB (2021) COVID-19 and the rule of law in Croatia: majoritarian or constitutional democracy? VerfBlog. 2021/4/27. https://verfassungsblog.de/covid-19-and-the-rule-of-law-in-croatia-majoritarian-or-constitutional-democracy/. 10.17176/20210427-101228-0

[CR63] Staunton C, Labuschaigne M (2021) COVID-19 in South Africa: a year in review. VerfBlog. 2021/3/11. https://verfassungsblog.de/covid-19-in-south-africa-a-year-in-review/. 10.17176/20210311-154106-0

[CR64] Syed S, Tariq-Ali N (2021) Democratic deficits of COVID-19 crisis in Pakistan: federal, regional and local response and coordination. VerfBlog. 2021/4/09. https://verfassungsblog.de/democratic-deficits-of-covid-19-crisis-in-pakistan/. 10.17176/20210409-101147-0

[CR65] Tagliabue F, Galassi L, Mariani P (2020) The "Pandemic" of disinformation in COVID-19. SN Compr Clin Med 1–3. 10.1007/s42399-020-00439-1 10.1007/s42399-020-00439-1 PMC7395797 32838179

[CR66] Tonsakulrungruang K, Leelapatana R (2021) Breeding more social turbulence?—Thailand’s Unprepared Response to the Second Wave of COVID-19. VerfBlog. 2021/2/23. https://verfassungsblog.de/breeding-more-social-turbulence-thailands-unprepared-response-to-the-second-wave-of-covid-19/. 10.17176/20210223-154114-0

[CR67] Uhlmann F, Ammann O (2021) Switzerland and the COVID-19 pandemic: a look back and a look into the future. VerfBlog. 2021/3/01. https://verfassungsblog.de/switzerland-and-the-covid-19-pandemic-a-look-back-and-a-look-into-the-future/. 10.17176/20210301-154022-0

[CR68] Vassileva R (2021) COVID-19 in autocratic Bulgaria: how the anti-corruption protests temporarily limited the abuse of questionable legislation. VerfBlog. 2021/3/05. https://verfassungsblog.de/covid-19-in-autocratic-bulgaria/. 10.17176/20210305-154130-0

[CR69] Vedaschi A (2021) COVID-19 and emergency powers in Western European democracies: trends and issues. VerfBlog. 2021/5/05. https://verfassungsblog.de/covid-19-and-emergency-powers-in-western-european-democracies-trends-and-issues/. 10.17176/20210505-111715-0

[CR70] Vikarská Z (2021) Czechs and balances—one year later. VerfBlog. 2021/3/30. https://verfassungsblog.de/czechs-and-balances-one-year-later/. 10.17176/20210330-195055-0

[CR71] Were N, Maleche A, Imalingat T (2021) COVID-19 in Kenya a year later: a case of Déjà Vu. VerfBlog. 2021/4/29. https://verfassungsblog.de/covid-19-in-kenya-a-year-later-a-case-of-deja-vu/. 10.17176/20210429-101233-0

